# The First GAEN-Based COVID-19 Contact Tracing App in Norway Identifies 80% of Close Contacts in “Real Life” Scenarios

**DOI:** 10.3389/fdgth.2021.731098

**Published:** 2021-11-17

**Authors:** Hinta Meijerink, Camilla Mauroy, Mia Karoline Johansen, Sindre Møgster Braaten, Christine Ursin Steen Lunde, Trude Margrete Arnesen, Siri Laura Feruglio, Karin Nygård, Elisabeth Henie Madslien

**Affiliations:** ^1^Department of Infectious Diseases and Vaccines, Norwegian Institute of Public Health, Oslo, Norway; ^2^Department of Infectious Diseases and Prevention, Norwegian Institute of Public Health, Oslo, Norway; ^3^Department of Information Technology (IT) Systems Bergen, Norwegian Institute of Public Health, Bergen, Norway; ^4^Center for Test and Quality Assurance at Norwegian Health Network, Norsk Helsenett, Trondheim, Norway; ^5^Norwegian Defence Research Establishment (FFI), Kjeller, Norway

**Keywords:** COVID-19, digital technology, mobile applications, contact tracing, exposure notification

## Abstract

The coronavirus disease 2019 (COVID-19) response in most countries has relied on testing, isolation, contact tracing, and quarantine (TITQ), which is labor- and time-consuming. Therefore, several countries worldwide launched Bluetooth-based apps as supplementary tools. The aim of using contact tracing apps is to rapidly notify people about their possible exposure to severe acute respiratory syndrome coronavirus 2 (SARS-CoV-2) and thus make the process of TITQ more efficient, especially upon exposure in public places. We evaluated the Norwegian Google Apple exposure notification (GAEN)-based contact tracing app Smittestopp v2 under relevant “real-life” test scenarios. We used a total of 40 devices, representing six different brands, and compared two different exposure configurations, experimented with different time thresholds and weights of the Bluetooth attenuation levels (buckets), and calculated the true notification rates among close contacts (≤2 m and ≥15 min) and false notification of sporadic contacts. In addition, we assessed the impact of using different operating systems and locations of the phone (hand/pocket). The best configuration tested to trigger exposure notification resulted in the correct notification of 80% of the true close contacts and incorrect notification of 34% of the sporadic contacts. Among those who incorrectly received notifications, most (67%) were within 2 m but the duration of contact was <15 min and thus they were not, *per se*, considered as “close contacts.” Lower sensitivity was observed when using the iOS operating systems or carrying the phone in the pocket instead of in the hand. The results of this study were used to improve and evaluate the performance of the Norwegian contact-tracing app Smittestopp.

## Introduction

Until March 9, 2020, all the cases of the coronavirus disease 2019 (COVID-19) in Norway were associated with travel or contact with a confirmed case (1). When cases with unidentified sources of infection were reported, Norway imposed comprehensive control measures, including the closure of schools, training facilities, and a variety of businesses and service industries. The response was based on “test, isolate, trace and quarantine (TITQ);” test suspected cases, isolate confirmed cases, identify and quarantine close contacts (2). One key factor of a successful TITQ strategy and breaking the chains of infection is the early identification of contacts. In 2020, Norway reported 50,130 confirmed cases, of which 31,155 were infected in Norway and 4,360 abroad (3). Based on the last 3 months of 2020, when the testing capacity allowed testing everyone with symptoms or suspected exposure, the source of exposure was unknown or missing in 20% of the reported cases (3, 4). This indicates that a significant proportion of cases and contacts were not identified through manual contact tracing or did not follow-up the advice given, as reported by other countries (5). With the introduction of new more contagious virus variants [variants of concern (VOC)] in winter 2021, the importance of efficient contact tracing strategies has been further stressed (6).

Manual contact tracing is an efficient tool in limiting the spread of COVID-19 but is labor-and time-consuming and depends on factors such as the capacity and experience of the local contact tracing teams, the number of contacts per confirmed case, as well as the quality of the information provided by the cases (7, 8). In Norway, contact tracing has sometimes been hampered by challenges including the hesitation to answer anonymous phone calls from the contact tracing teams, lacking or incorrect contact information (e.g., people with temporary citizenship or tourists), language/cultural barriers, gaps in memory, or general unwillingness to collaborate. Therefore, contact notification might be delayed by days and some contacts might not be identified. Digital solutions, such as Bluetooth-based apps, have been proposed as supplementary tools and have the advantage of providing rapid notification of possible community exposures (9–11). This could be particularly helpful in hot-spot areas, where the population density is high and social distancing in public spaces is challenging. Furthermore, the introduction of more contagious virus variants, such as the alpha and delta variants, has clearly shown how rapid outbreaks may evolve, causing even more stress on the local contact tracing teams. Thus, digital apps could be a valuable tool in the fight against the pandemic. However, the Bluetooth-based technology, which most of these tools rely on, still has several limitations in terms of the ability to correctly identify close contacts (12). Proximity estimation is based on the decaying/attenuation of Bluetooth signal, measured as the attenuation of the Received Signal Strength Indicator (RSSI) values (dBm), which is affected by many factors and does not offer precise measurement of distance (12, 13). Furthermore, the success of the app strongly depends on the acceptance of the population to use it. App acceptance and efficient use depend on many factors such as technology, architecture, communication by public authorities, and cultural context (14, 15).

In April 2020, Norway released the COVID-19 contact tracing app (Smittestopp v1), which used a centralized approach that registered the data of Bluetooth contacts and locations into a central database. This solution was intended to fulfill two purposes: notifying the close contacts of individuals with confirmed COVID-19 infection and analyzing movement patterns and population behavior during the pandemic. However, in June 2020, Smittestopp v1 was shut down by the Norwegian Data Protection Authority (Datatilsynet) due to privacy concerns, specifically regarding the centralized storage of positional data and Bluetooth contacts. Subsequently, Norway developed a new app (Smittestopp v2) based on the Google/Apple Exposure Notification (GAEN) application programming interface (API), which uses a decentralized architecture and allows international integration (16, 17). The Smittestopp v2 was launched in December 2020 and as of September 12, 2021, was downloaded more than 1 million times and ~5,000 out of the total 176,000 infections were reported (18). This gives a download rate of ~23% among smart-phone users in the age group 9–79 years and 18.5% of the total Norwegian population, which is lower than those reported from similar apps in neighboring countries (Finland/ “Koronavilkku:” 45%; Denmark/ “Smittestop:” 38%) (19).

Over 20 countries have implemented GAEN based contact tracing apps, including Germany, Ireland, Denmark, Netherlands, Switzerland, Canada, and Japan (20). Decentralized apps do not collect information to protect privacy, making the evaluation of the app challenging, and other sources are therefore needed. In Switzerland, 41 out of 6,380 (0.6%) confirmed cases reported receiving a notification in the app (SwissCovid; launched June 2020) as the reason for testing (21). The app from Denmark (Smitte|stop) has been downloaded over 2 million times and with 67,000 infections reported since June 2020 (22).

It is essential to test, determine, and optimize the ability of contact tracing apps upon release. Our study compared the notification rates among close and sporadic contacts using two different configurations of the Norwegian GAEN-based contact tracing app Smittestopp v2. By adjusting the settings for triggering a notification, we aimed at achieving a correct notification rate of at least 75% for close contacts under “real-life” scenarios.

## Materials and Methods

### Devices Used for Testing

To test the GAEN configurations, we used 40 phones with an even mix of Android and iOS operating systems represented by different brands and models available on the Norwegian market (23). A development (beta) version of the app was used to allow data collection on each phone, as well as the use of artificial identification (ID)-keys [temporary exposure keys (TEKs)] to verify positive COVID-19 test results.

### Test Scenarios

**Figure 1:**
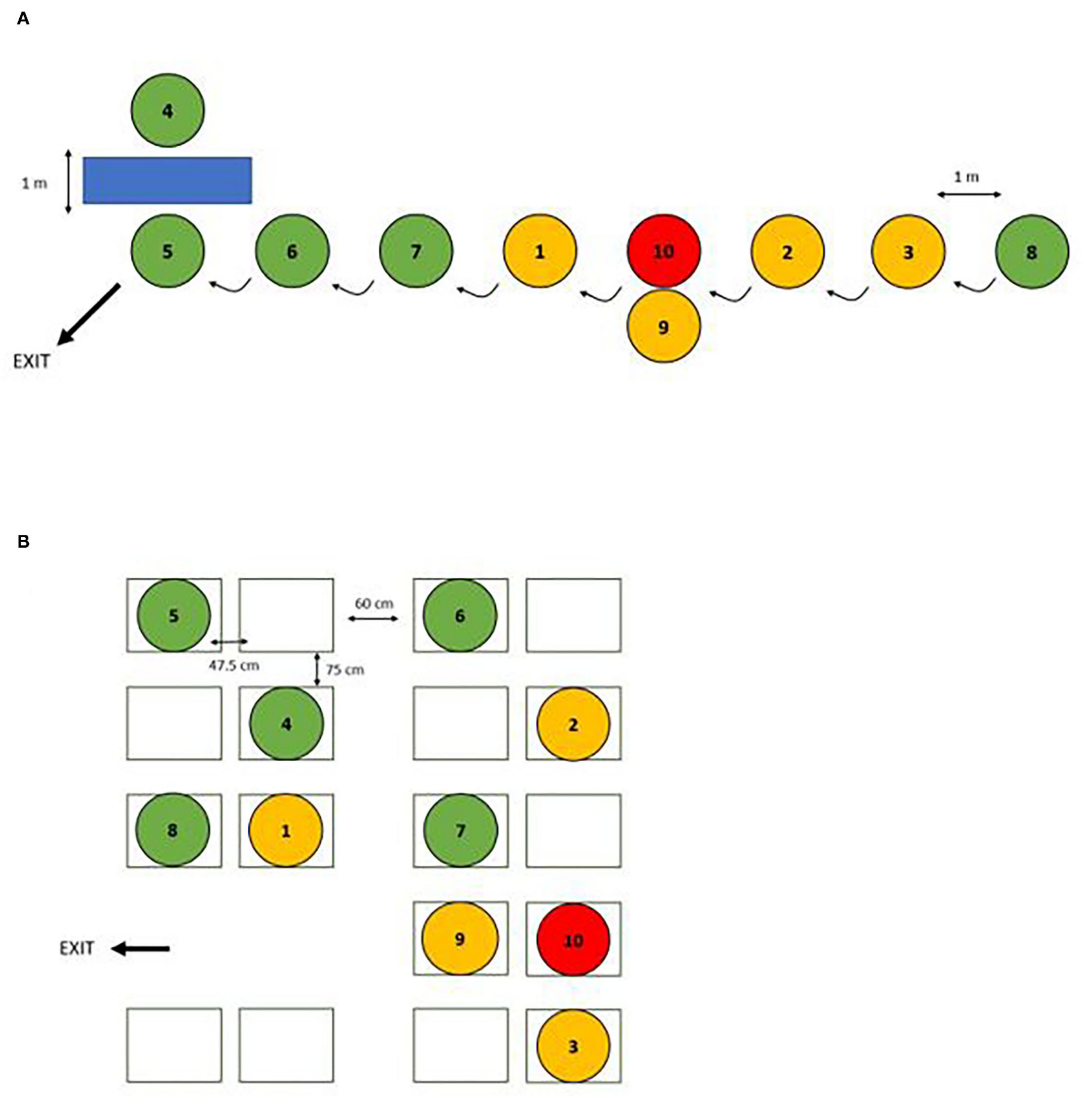
Schematic representation of the **(A)** “queue” scenario, where participants simulated standing in a queue, and **(B)** “bus” scenario, where participants simulated taking a bus to evaluate the notification rates *via* contact tracing under various configuration settings. Red: “infected"; orange: “close contact"; green: “sporadic contact”.

We set up simulations of two scenarios where people are likely to be nearby strangers in real-life situations, namely, standing in a supermarket queue and traveling with public transport (Figure 1). All the tests were performed in a closed room with no windows. Both scenarios included 10 participants who each carried two phones. All the phones were fully charged before the experiments, but the battery charge level at end of each experiment was not controlled. All participants were instructed to carry one phone in their hand and put one phone in their pocket. Of the 10 participants, one was defined as “infected,” four were defined as “close contacts” (≤2 m for ≥15 min), and five were defined as “sporadic contacts” (either 3–4 m away or ≤2 m for <15 min). In the queue scenario, one participant was situated in the position of a cashier and all other participants were positioned 1 m apart in a queue. In the bus scenario, we used the measurement of city buses as a guide and simulated a bus ride. For both scenarios, the participants were instructed to take different positions at predefined time intervals. The total duration of each scenario was 20 min and each experiment (queue and bus) was repeated at least once with each configuration setting to control the variability between runs and ensure sufficient data points. A positive control, being constantly closer than 1 m to the infected phone, was included in each run. The detailed test protocol, including an overview of the different settings, can be shared with interested parties upon request.

### Data Collection

After each run, we activated the notification function of the app on the “infected” device wherein the exposure data was pulled on the “exposed” devices and manually entered into Excel spreadsheets (Microsoft Office, Microsoft, Redmond, Washington, United States). Before each test run, all previous TEKs and exposure data were deleted from each of the devices used in the experiments.

### Defining Attenuation Thresholds (Configurations)

Two different configurations (#1 and #2) were selected for our test protocol (Table 1). These were based on internal test reports of configurations used in the Danish app Smitte|stop as well as test reports from similar apps used in other countries. Each configuration was defined by different threshold levels (“buckets”) of Bluetooth signal attenuation (dB) that would be registered as exposure. We used three different buckets based on the attenuation thresholds, “low,” “medium,” and “high.” The “low” bucket has the lower attenuations and we used this as a proxy for people who are close by, assuming that an exposure closer than 1 m would primarily be registered in this bucket and that attenuation would increase with distance. We used the “medium” bucket as a proxy for those relatively further away (1–2 m) but likely still within relevant range for exposure and the “high” attenuation threshold was set so that people registered in this bucket were not relevant for exposure (>2 m).

**Table 1:** Overview of the configurations^*^ used in the scenario-based testing.

**Bluetooth signal attenuation level (“bucket”)**	**Expected relative distance between devices**	**Configuration #1**	**Configuration #2**
Low	Short (<1 m)	<57 dB	<57 dB
Middle	Medium (1–2 m)	57–63 dB	57–68 dB
High	Long (>2 m)	>63 dB	>68 dB

* *The low bucket configuration thresholds are set with the aim to detect contacts being closer than 1 m, the middle bucket to detect contacts being 1–2 m away, and the high bucket is set to detect contacts further than 2 m away*.

### Balancing Notification Rates Among Close and Sporadic Contacts

We aimed at achieving a high notification rate (at least 75%) of “true” close contacts (≤2 m for ≥15 min) and at the same time, minimizing the risk of “false” notification of sporadic contacts. Consequently, we assessed the impact of using different weights for the time spent in each of the buckets (range 0–2.5) as well as different time thresholds (range 10–15 min) that would trigger a notification. A total of 15 unique settings (combination of weights and time thresholds) were assessed for each of the two test configurations (#1 and #2). The configurations that were tested included the following combinations:

- Low bucket/Weight 2.5-Middle bucket/Weight 1- Time threshold 10/13/15 min
- Low bucket/Weight 2.5- Middle bucket/Weight 1.5- Time threshold 10/13/15 min
- Low bucket/Weight 2.5- Middle bucket/Weight 2.0- Time threshold 10/13/15 min
- Low bucket/Weight 2- Middle bucket/Weight 1.0- Time threshold 10/13/15 min
- Low bucket/Weight 2- Middle bucket/Weight 1.5- Time threshold 10/13/15 min

Notifications would be sent to all phones where the cumulative weighted times of all the detected exposures were above these thresholds. For example, weights of 1.5 (low) and 1 (middle) with a time threshold of 15 min would result in the time registered in the low bucket counting 1.5 time and the time registered in the middle bucket counting 1 time. When the weighted sum of the time is above 15 min, a person will receive a notification. This means that when a phone registers proximity to a confirmed case of 10 min in the medium bucket and 4 min in the low bucket, these weights would result in a proximity registration of 16 min total and therefore trigger a notification message. Note that the time in the highest bucket (Table 1) was not counted in any of the settings. The settings that were found to give the highest notification rate of close contacts were subject to further analyses to evaluate the impact of different scenarios, operating systems, and locations of the device (hand vs. pocket). We calculated the proportion of the phones receiving notifications among close contacts and sporadic contacts and used a chi-square test to look at the differences and used Cohen's power test to calculate the statistical power. Based on the assumption of 75% notifications among close contacts and 30% among sporadic contacts, we would have a power of 80% for 95% confidence in our results with 16 phones per group. All data analyses were performed in Stata/SE 16.0 (StataCorp LLC, Texas, United States).

## Results

The relatively low sample size of this study implies that the results must be interpreted with caution.

### Configuration #1

Overall, among the close contacts, 69% of the devices registered time in the low (<57 dB) or middle (57–63 dB) attenuation buckets, compared with 45% among sporadic contacts. The highest notification rate of true close contacts was found to be 62–63%. At the same time, the false notification of sporadic contacts was found to be 23–31%. Using a time threshold of 10 min, instead of 13 or 15 min, resulted in higher notification rates, regardless of the weights given to each bucket. The three experimental settings that were found to give the highest accuracy were selected for further analyses to assess the impact of different operating systems and the carriage of the device. The results indicate that notification rates would be significantly (*p* < 0.05) lower when using the iOS operating system compared with Android (Table 2). Furthermore, carrying the device inside pockets instead of in the hand would significantly (*p* < 0.05) decrease the sensitivity of the app. Among the sporadic contacts, the notification rates were significantly higher among those who were within 2 m for <15 min compared with those further (2–4 m) away (*p* < 0.01). Thus, incorrect notifications were mostly sent to people who were very close (<2 m) for a shorter duration than 15 min. We found no significant differences in the notification rates between the two scenarios (queue and bus).

**Table 2:** Notification rates among close contacts and sporadic contacts using configuration #1 with selected weights split by various variables.

**Setting (weights of buckets and time thresholds)**	**Variable**		**Close contacts** **% (*n*)**	***p*-value** **(chi-square)**	Sporadic contacts^*^ **% (*n*)**	***p*-value** **(chi-square)**
**1.1**						
	**Scenario**			0.538		0.228
**Low:**		Queue	58 (19)		29 (11)	
2.5		Public transport	64 (29)		18 (8)	
**Middle:**	**Operating system**			0.026		0.034
1.0		iOS	49(18)		13 (5)	
**Time:**		Android	73 (30)		33 (14)	
10 min	**Location phone**			0.494		0.016
		Hand	66 (23)		34 (14)	
		Pocket	58 (25)		12 (5)	
**1.2**						
	**Scenario**			0.729		0.319
**Low:**		Queue	61(20)		37 (14)	
2.5		Public transport	64 (29)		27 (12)	
**Middle:**	**Operating system**			0.046		0.002
2.0		iOS	51 (19)		47 (20)	
**Time:**		Android	73 (30)		15 (6)	
10 min	**Location phone**			0.343		0.001
		Hand	69(24)		49 (20)	
		Pocket	58 (25)		14 (6)	
**1.3**						
	**Scenario**			0.538		0.228
**Low:**		Queue	58(19)		29 (11)	
2.0		Public transport	64 (29)		18 (8)	
**Middle:**	**Operating system**			0.026		0.034
1.5		iOS	49(18)		13 (5)	
**Time:**		Android	73 (30)		33 (14)	
10 min	**Location phone**			0.494		0.016
		Hand	66(23)		34 (14)	
		Pocket	58 (25)		12 (5)	

* *Sporadic contacts are those that do not fulfill the criteria of the “close contact”-definition; ≤ 2 m for ≥15 min*.

### Configuration #2

Overall, among the close contacts, 94% of the devices registered time in the low (<57 dB) or middle (57–68 dB) attenuation buckets, compared with 68% of the sporadic contacts. The highest notification rate of true close contacts was found to be 89%, which unfortunately also resulted in a high notification rate (55%) of sporadic contacts. The three experimental settings that were found to give the highest accuracy were subject to further analyses to assess the impact of different operating systems and carriage of the device, as described for configuration #1 in Section 3.1. In general, the durations of 13 and 15 min seemed to give higher accuracy compared with 10 min, as opposed to configuration #1. Similar to configuration #1 we found a lower sensitivity when using the iOS operating system, although the evidence was weaker (*p* = 0.06) than with configuration #1 (*p* < 0.05) (Table 3). Carrying the phone in the pocket gave significantly (*p* < 0.01) lower sensitivity, similar to configuration #1. We found no significant differences in the notification rates between the two scenarios (queue and bus). Among the sporadic contacts, the participants who were within 2 m for <15 min had a significantly higher notification rate (*p* < 0.01) than those being further (3–4 m) away.

**Table 3:** Notification rates among close contacts and sporadic contacts using configuration #2 with selected weights split by various variables.

**Setting (weights of buckets and time thresholds)**	**Variable**		**Close contacts** **% (*n*)**	***p*-value** **(chi-square)**	Sporadic contacts^*^ **% (*n*)**	***p*-value** **(chi-square*)***
**2.1**						
	**Scenario**			0.612		0.732
**Low:**		Queue	77 (13)		32 (6)	
2.5		Public transport	83 (15)		37(7)	
**Middle:**	**Operating system**			0.062		0.087
1.5		iOS	68 (13)		21 (4)	
**Time:**		Android	94 (15)		47 (9)	
13/15^**^ min	**Location phone**			0.042		0.207
		Hand	94 (16)		44 (8)	
		Pocket	67 (12)		25 (5)	
**2.2**						
	**Scenario**			0.330		0.740
**Low:**		Queue	77 (13)		42 (8)	
2.5		Public transport	90 (16)		37 (7)	
**Middle:**	**Operating system**			0.117		0.020
2.0		iOS	74 (14)		21(4)	
**Time:**		Android	94 (15)		58 (11)	
15 min	**Location phone**			0.009		0.054
		Hand	100 (17)		56 (10)	
		Pocket	67 (12)		25 (5)	
**2.3**						
	**Scenario**			0.612		0.732
**Low:**		Queue	77 (13)		32 (6)	
2.0		Public transport	83 (15)		37 (7)	
**Middle:**	**Operating system**			0.062		0.087
1.5		iOS	68 (13)		21 (4)	
**Time:**		Android	94 (15)		47 (9)	
13 min	**Location phone**			0.042		0.207
		Hand	94 (16)		44 (8)	
		Pocket	67 (12)		25 (5)	

* *Sporadic contacts are those that do not fulfill the criteria of the “close contact”-definition; ≤ 2 m for ≥ 15 min*.

** *Time thresholds of 13 and 15 min generated identical results*.

## Discussion

In this study, we aimed at optimizing the precision of the GAEN-based contact tracing app Smittestopp v2 under “real-life” scenarios to target close contacts. Although this study is limited by a relatively small sample size, our results indicate that the performance of the app could be considerably improved by adjusting the settings. This included the Bluetooth attenuation levels, time thresholds for generating an alert, and weight of time registered in each attenuation level. As expected, we observed variations between different devices, with the iOS operating system generally having a lower sensitivity than Android. Similar findings were observed when the device was carried in the pocket. This supports previous knowledge on the inaccuracy of Bluetooth-based proximity estimation, and this is an important aspect to take into consideration when communicating advice to the public (12, 24).

The success of Smittestopp as a tool to control national outbreaks relies on its ability to timely and correctly identify and notify those exposed as well as having a high adoption rate in the population. Although difficult to model, others have suggested that an adoption rate of at least 20% already has an impact on infection rates (25). Presumably, the benefit of contact tracing apps would be greatest in areas where the incidence rate is high, the prevalence of undetected cases is high, and in times and areas where the capacity of contact tracing teams is exceeded. However, since the local storage of data in Smittestopp does not allow the identification and direct follow-up of cases and contacts, the app can only function as a supplement to, and not replace, manual contact tracing conducted by the local health authorities. Thus, to maximize the benefit of this tool, it is important to target those who might get exposed outside their household in public spaces where manual contact tracing is particularly challenging (26). Furthermore, it is important to reach subgroups of the public (e.g., younger age groups and immigrants) who have been overrepresented among COVID-19 cases and living in densely populated areas where the incidence rate has remained relatively high during the pandemic (27).

Many countries have implemented contact tracing apps based on the GAEN solution, but their effectiveness to control the COVID-19 pandemic (e.g., in terms of reducing R_eff_, and local incidence rate) have been questioned, particularly due to the low level of population uptake and the inaccuracy of the Bluetooth technology in proximity estimation (28). Studies from the United Kingdom, which have implemented the GAEN-based National Health Service (NHS) COVID-19 app along with manual tracing, provided evidence and support for the epidemiological benefits and continued implementation of digital tracing apps until the population has been protected through vaccination (29, 30). Regarding the technical performance of such apps, the lack of harmonized terminology and procedures for testing and evaluation makes it difficult to compare test results across countries. At the time when the Norwegian app Smittestopp v2 was developed in fall 2020, very few countries had published data on the technical performance of their apps. Thus, we mostly had to rely on technical reports that were available on the web as well as information collected through direct communication with the health authorities in other countries. The results of the scenario-based testing of the German app (Corona-warn) were presented on GitHub, showing that the notification rates among close contacts were 47% with bucket thresholds of 55 and 63 dB (31). These results are in line with the findings in the current study using configuration #1 settings. Furthermore, the Netherlands reported on a field testing with seven scenarios to decide the optimal settings to identify exposure within 1.5 m for at least 15 min. These tests identified that the cut-off value should be between 68 and 75 dB and the time threshold should be <15 min for closer contacts. Therefore, they included all contacts with an attenuation ≤63 dB for >10 min, or 64–73 dB for >15 min (32). Considering that the Netherlands uses risk scores instead of buckets, meaning they will identify exposure based on single contacts, these results are in line with the settings chosen in Norway. On the other hand, Switzerland reported a configuration that is stricter than in Norway; low bucket ≤ 55 dB weighted 1, medium bucket 55–63 dB weighted s0.5, and a time threshold of 15 min over 1 calendar day. They showed that 88% of phones within 2 m had an attenuation below 63 dB, but it is unclear how the duration of exposure was factored in (33). Our study adds to the current literature by showing the influence of changing the configuration settings on the notification of close contacts and sporadic contacts, the considerations taken into account when choosing the settings, as well as some of the factors affecting exposure identification such as the operating system of the phone and the location of the phone during exposure by using real-life scenarios with different phones. However, more work needs to be done to define the configuration standards and harmonize the testing and evaluation protocols across countries.

In Norway, contact tracing is normally initiated to identify and quarantine those who have been within 2 m for over 15 min to a confirmed COVID-19 case during the infectious phase of the disease (2). However, the true risk of being infected depends on a combination of factors related to the host, the exposed, the exposure situation, and the environment as well as the virus itself (34). During manual contact tracing, health care professionals evaluate the risk and will recommend quarantine and/or testing based on this assessment. With digital apps, such as Smittestopp, notifications need to be sent based on a set of criteria defined by the app, namely duration of contact, Bluetooth attenuation, and when exposure took place. Due to Bluetooth attenuation, as well as the duration, being affected by many factors, these digital contact tracing apps are not specific in sending notifications to close contacts only. The decisions on the configuration settings will therefore always be a balance between sending notifications to “true” close contacts and limiting the notifications sent to sporadic, low-risk contacts, as well as the subsequent recommendations given to the notified, which can be affected by the testing capacity. The decision on the current configuration settings was based on a combination of variables; (1) notification rate among close contacts, (2) notification rate among sporadic contacts, and (3) the type of advice and measures for contacts. We adjusted the thresholds of the buckets to achieve a correct notification rate of a minimum of 75%. In addition, closer proximity was deemed a higher risk factor than duration and therefore, we accepted higher notification rates for those within 2 m for <15 min than among those further away. Recommending quarantine for contacts identified *via* the app was not considered to be a proportionate measure, based on the known inaccuracy of the Bluetooth technology as well as the inability of the app to identify the last date of exposure. In Norway, people identified as contacts through the app have been advised to get tested and stay home until the test result is ready. Thus, choosing settings that would allow a certain percentage (30–40%) of “non-close,” sporadic contacts to be notified was considered acceptable. Based on the overall testing capacity from December 2020 to May 2021, people have been able to get tested and receive their results within 24–48 h in most of the country. The high level of data protection makes it difficult to estimate the added benefit of implementing the app, compared with relying on manual contact tracing only. Results from a survey in Oslo indicate that around 30% of the cases that tested positive in March 2021 had downloaded the app (35). Among these, 12% reported that they had received a notification of exposure through the app. However, only 42% remembered to report themselves as infected through the app, identifying an important area for improvement for the app to be effective.

In conclusion, we showed that the accuracy of the app could be considerably improved by adjusting the GAEN-configurations. These findings provide guidance to health authorities on the expected notification rates and limitations of app-based contact tracing. Experimental data on the performance of the app under real-life conditions could help build confidence among the public as well as push the technological processes and improvement forward. These configurations are easily adjustable and should be regularly reassessed based on a combination of factors that could change over time, such as disease prevalence, increased transmissibility of new virus variants (e.g., VOCs), as well as changes in national advice and control measures. Thus, the configurations settings should be carefully adapted to the national situation and tested under relevant exposure scenarios and not copied from other existing solutions abroad. Although there are still technological and other limitations that need to be overcome before GAEN-based apps could replace manual contact tracing, we believe that transparency around the development and testing could contribute to the increased acceptability and trust among the public.

## Data Availability Statement

The raw data supporting the conclusions of this article will be made available by the authors, upon request.

## Ethics Statement

Ethical review and approval was not required for the study on human participants in accordance with the local legislation and institutional requirements. Written informed consent for participation was not required for this study in accordance with the national legislation and the institutional requirements.

## Author Contributions

HM and EM: study design, data collection, data analysis, data interpretation, and writing. CM and MJ: data collection, data interpretation, and writing. SB, TA, SF, and KN: study design, data interpretation, and writing. CL: study design, data collection, and writing. All authors contributed to the article and approved the submitted version.

## Funding

The Norwegian Institute of Public Health (NIPH) funded this project as part of the development of the Norwegian digital contact tracing app (Smittestopp v2).

## Conflict of Interest

The authors declare that the research was conducted in the absence of any commercial or financial relationships that could be construed as a potential conflict of interest.

## Publisher's Note

All claims expressed in this article are solely those of the authors and do not necessarily represent those of their affiliated organizations, or those of the publisher, the editors and the reviewers. Any product that may be evaluated in this article, or claim that may be made by its manufacturer, is not guaranteed or endorsed by the publisher.
